# A Preliminary Investigation into *Penicillium* spp. Growth on Peanuts During Drying and Storage

**DOI:** 10.3390/life15020140

**Published:** 2025-01-21

**Authors:** Daniela Campaniello, Annalisa d’Amelio, Angela Guerrieri, Alessandra Accettulli, Alessandro De Santis, Antonio Bevilacqua

**Affiliations:** Department of Agriculture, Food, Natural Resources and Engineering (DAFNE), University of Foggia, Via Napoli 25, 71122 Foggia, Italy; daniela.campaniello@unifg.it (D.C.); annalisa.damelio@unifg.it (A.d.); angela.guerrieri@unifg.it (A.G.); alessandra.accettulli@unifg.it (A.A.); alessandro.desantis@unifg.it (A.D.S.)

**Keywords:** fungi, variety, inoculum level, spore age, temperature, duration

## Abstract

Fungal contamination represents a significant threat during peanut storage. In this research, a strain of *Penicillium* spp. was used as a test microorganism to assess its viability during peanut storage over 30 days at three different temperatures (4, 15, and 25 °C) and at two different inoculum levels (low-2 log CFU/g and high-5 log CFU/g). Two peanut types were tested: the Spanish type and the Virginia type. Independently of spore age, the fungus survived throughout the storage period, and in some samples (low inoculum Virginia-type peanuts) its level increased. In the second phase, four drying treatments, differing in temperature and duration, were tested. Fungal inactivation primarily depended on the temperature, while the duration of the drying process did not have a significant effect. At low temperatures, fungal inactivation was minimal and not statistically significant, suggesting that low-temperature treatments could pose a potential health risk.

## 1. Introduction

The peanut (*Arachis hypogaea* L.), also known as the groundnut or American peanut, is an allotetraploid (AABB genome) and belongs to the *Arachis* section within the family Leguminosae [1]. Botanically, it is a caespitose annual plant that grows to a height of 40–60 cm. After fertilisation, the flower peduncle elongates, and due to positive geotropism, the ovary penetrates to a depth of 5–15 cm, where the legume develops and matures [2]. The fruit of the groundnut consists of an indehiscent, reticulate, tuberous, oblong legume with more or less pronounced constrictions, enclosing seeds that are cylindrical to globose in shape and can vary in number from one to four [2].

Groundnuts are primarily cultivated in tropical and temperate regions, with an annual production of approximately 39.9 million tons. The leading producers include the United States, Argentina, Sudan, Senegal, and Brazil [3,4]. As the thirteenth most important food crop globally, peanuts play a significant role in the global economy and, like all food products, require stringent monitoring from a food safety perspective [5]. Unfortunately, the nutritional value of groundnuts is significantly compromised by mycotoxin contamination, particularly during the post-harvest phase [3]. Peanuts, being nutrient-rich, especially their seeds, serve as an ideal substrate for fungi belonging to the genera *Rhizopus*, *Penicillium*, and *Aspergillus*. These fungi can produce mycotoxins, resulting in necrosis, seed rot, wilting, grey and black mould, and leaf spots, leading to substantial yield losses in both quality and quantity [3].

Contamination can occur at various stages: pre-harvest; in the field when the fruit is still on the plant; or during ripening, when fungal spores can contaminate the fruit through insect damage. Post-harvest contamination can also occur during peeling, washing, and sorting [6]. These steps are critical and must be carried out meticulously, with washing being the most critical stage, as the moisture makes the product highly susceptible to fungal growth and mycotoxin development. Mycotoxins represent a severe health hazard, as the release of carcinogenic toxins, if not detected in time, can reach the consumer and cause lung or liver cancer, as well as cardiovascular diseases [4,7,8]. Another step that may lead to contamination issues is improper storage, particularly if conducted under unsuitable temperature and humidity conditions [3]. Given the widespread use of peanuts, the development of mycotoxins, especially aflatoxins, poses a significant global health concern as they can cause chronic toxicity (e.g., cirrhosis of the liver) and are hepatocellular carcinogens [9]. Additionally, lipid oxidation in groundnut meal is one of the primary causes of spoilage due to the degradation of fatty acids, particularly linoleic and linolenic acids. Lipid oxidation leads to reduced shelf life, off-flavours, nutrient loss, and the development of undesirable aromas during storage of groundnut flour [10].

The objectives of this study are to present a case study assessing the growth potential of *Penicillium* spp. on peanuts, monitor fungal development, and evaluate the safety and stability of peanuts during storage. Furthermore, a viability test was conducted during different drying processes to determine whether fungi could survive these treatments.

## 2. Materials and Methods

### 2.1. Fungal Spore Production

A fungal strain of *Penicillium* spp., belonging to the culture collection of the Predictive Microbiology Group of the DAFNE Department of the University of Foggia, was used in this study.

A permanent stock of the fungal strain was stored at 4 °C on Potato Dextrose Agar (PDA) (Oxoid, Milan, Italy). To prepare a conidial suspension, mycelial fragments were transferred onto PDA and incubated at 25 °C for 7 days. The conidia were then harvested by washing the surface of the plate with 10 mL of a Tween 80 solution (0.05% *v*/*v*) (J.T. Baker, Milan, Italy). Briefly, the spores were collected from the agar by flooding the culture with the Tween 80 solution and dislodging the spores from the hyphae using a sterile glass spreader. After preparation, the conidial suspension was serially diluted in saline solution (0.9% NaCl), inoculated onto the surface of PDA plates (100 µL per plate), and spread evenly using a sterile glass spreader. The plates were incubated at 25 °C for 5 days.

### 2.2. Penicillium Growth on Peanuts

The survival of *Penicillium* spp. was evaluated using two peanut genotypes, the Virginia and Spanish types, which were analysed at different temperatures using both freshly produced spores (CS_0_) and spores pre-stored at 4 °C for 30 days (CS_30_).

Twenty grams of peanut (moisture content: 30%) was placed into sterile Petri dishes and inoculated at two different levels (low inoculum: 2 log CFU/g and high inoculum: 5 log CFU/g) by distributing 1 mL of a conidial suspension (at 7 log CFU/mL for high inoculum and 4 log CFU/mL for low inoculum conditions) using a pipette. The samples were then stored at 4, 15, and 25 °C, and periodically analysed to determine the level of penicillia.

For analysis, 10 g of peanuts was mixed with 90 mL of a sterile saline solution (0.9% NaCl solution) in a Stomacher bag (Seward, London, UK) and homogenized for 1 min using a Stomacher Lab Blender 400 (Seward). Serial dilutions of the homogenates were then distributed onto PDA plates, which were incubated at 25 °C for 3–5 days. Each assay was performed in triplicate.

### 2.3. Drying

The samples were then subjected to three different drying cycles in an oven, with the specific combinations outlined in Table 1. Peanuts were inoculated at 7 log CFU/g, and after treatment, the concentration of surviving spores was determined through viable counts on PDA, as described above.

### 2.4. Statistics

All experiments were performed at least in duplicate over two different batches (different sample sets), and each batch was analysed twice (replicated analyses).

Significant differences were identified using one-way analysis of variance (ANOVA), with Tukey’s test employed as a post hoc comparison test (*p* < 0.05). Linear regression was also conducted to evaluate significant correlations during the drying experiment.

Statistics were recorded using Statistica for Windows software, version 7.0 (Statsoft, Tulsa, OK, USA).

## 3. Results

The first step aimed to investigate spore survival during storage at two different inoculum levels. The low inoculum condition was selected based on the limit of detection (LOD) of the spread plate method (2 log CFU/g), while the high inoculum level was determined from preliminary experiments that indicated that no inhibitory phenomena occurred at 5 log CFU/g. Table 2 and Table 3 present the concentrations of *Penicillium* spp. during the storage of peanuts. Irrespective of temperature, the fungus survived for at least 30 days in both samples inoculated with freshly produced spores (CS_0_) and pre-stored spores (CS_30_).

At low inoculum levels (2 log CFU/g), as shown in Table 4 and Table 5, an increase in fungal concentration was observed for certain combinations and sampling times, particularly at 25 °C and primarily with freshly produced spores. This suggests that *Penicillium* spp. proliferation could occur during peanut storage under these conditions.

**Figure 1:**
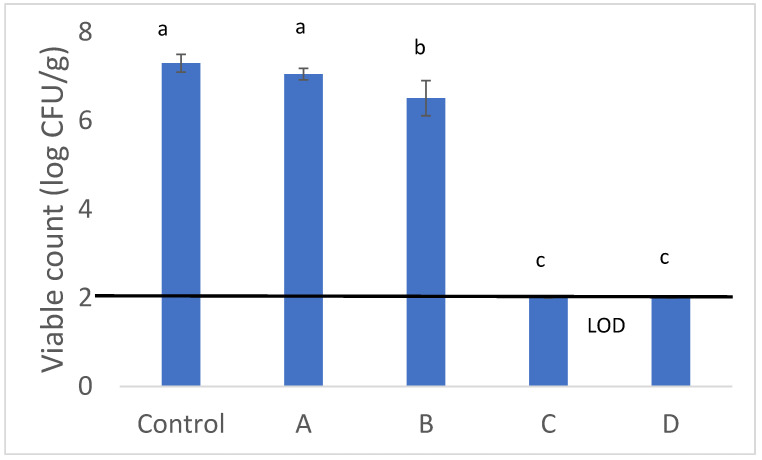
The viable counts of *Penicillium* after the drying treatments. Mean values ± standard deviation are shown. For treatments, see Table 1. Different letters indicate a significant difference among the samples (*p* < 0.05; ANOVA and Tukey’s test). LOD, limit of detection.

The second step of this research focused on another critical aspect of peanut shelf life and consumption: drying. The process, as suggested by some of the literature, was carried out using different time/temperature combinations. Figure 1 shows the viable counts of penicillia after the drying treatments. In sample A, a viable count of 7.05 log CFU/g was observed, compared to 7.30 log CFU/g in the control. In combination B, the viable count decreased to 6.51 log CFU/g, representing a reduction of 1.8 log CFU/g compared to the control. Finally, in combinations C and D, the penicillia were reduced to below the LOD, achieving a reduction of at least 5 log CFU/g.

The data of the viable counts of penicillia after the drying process were used as input values for a regression model to assess the quantitative effects of time and temperature on spore counts following the treatment. The model’s output includes the mathematical coefficients, the P-value, and the R^2^ coefficients, as shown in Table 6. The results suggest that, at least for the combinations tested in this study, temperature is the primary parameter influencing fungal survival, while time is not statistically significant. It is important to note that the regression analysis was performed solely using the data collected in this research. Therefore, the actual role of the treatment duration should be further clarified through confirmatory experiments.

## 4. Discussion and Conclusions

Fungi, particularly *Aspergillus* spp. and *Penicillium* spp., are significant causes of concern in peanut storage [8,11]. These fungi produce mycotoxins, especially aflatoxins, which are highly toxic and carcinogenic secondary metabolites that pose serious health risks to humans [9]. This study aimed to optimize post-harvest processes, with a particular focus on storage conditions, including humidity and temperature. The research was carried out on two peanut cultivars, Virginia and Spanish types, using *Penicillium* spp. as reference microorganisms and their spores as targets.

The storage tests revealed that both cultivars were susceptible to fungal colonization and growth, regardless of temperature conditions. This was evident for both high and low concentrations, as well as for freshly produced and pre-stored spores. Although we found that, at a low inoculum, some samples showed concentrations of penicillia below the LOD, this output was likely due to uneven inoculum distribution on the samples rather than an actual inhibitory phenomenon.

Since peanuts are not dried immediately upon harvest, but rather just before consumption, and given the presence of old spores in the environment (and consequently during storage), this situation poses a significant health risk. Under favourable conditions, the fungus can proliferate on the stored peanuts [12,13]. Peanuts with a moisture content of 10% or higher are particularly vulnerable to mycotoxin contamination, emphasizing the importance of proper storage practices. Effective control of mycotoxin contamination during the post-harvest phase requires timely drying and maintenance of safe moisture levels [3,14].

Peanut drying can be done using various temperatures (from 35 to 60–65 °C) and durations [15], as it has been suggested that low-temperature processes may delay lipid oxidation [15,16]. However, to the best of our knowledge, there are limited data on the effects of different drying processes on fungi survival during and after the treatment. Therefore, some combinations were selected based on evidence from the literature [15,17] and tested in this study. A multiple regression analysis of the drying test results demonstrated that the differences in fungal concentration were significantly influenced by temperature. Specifically, higher temperatures led to substantial reductions in fungus levels, with a 5-log decrease observed in treatments C (55 °C) and D (60 °C). As previously mentioned, partial replacement of high-temperature processes has been proposed as a strategy to counteract lipid rancidity [16]. However, while these treatments may address lipid oxidation, they do not fully inactivate fungi, which could pose health risks.

In conclusion, this study highlights the potential for fungal survival during peanut storage under both ambient temperatures and refrigerated conditions across two different varieties, regardless of the type of contamination (fresh or old spores). Additionally, the results indicate that temperature plays a crucial role in the inactivation of fungi during drying, whereas the impact of treatment duration requires further investigation, particularly in relation to its effects on fatty acids and the lipid oxidation of peanuts. Furthermore, the use of low-temperature cycles poses a significant health risk, as fungal inaction may be minimal or not significant.

## Figures and Tables

**Table 1:** Conditions of treatment drying cycles.

Treatment	Temperature (°C)	Time (h)
A	35	18
B	45	10
C	55	6
D	60	48

**Table 2:** *Penicillium* spp. during the storage of peanuts (Virginia type) at 4, 15, and 25 °C (log CFU/g), high inoculum conditions. The samples were inoculated with freshly produced spores (CS_0_) or with spores preliminarily stored at 4 °C for 30 days (SC_30_). For each column, the letters indicate significant differences among the sampling times; the letter a represents the lowest value, and the alphabetical order indicates the values’ increase (ANOVA and Tukey’s test, *p* < 0.05).

	25 °C		15 °C		4 °C	
Storage Time (Days)	CS_0_	CS_30_	CS_0_	CS_30_	CS_0_	CS_30_
0	5.00 ± 0.04 ^a^	5.00 ± 0.04 ^b^	5.00 ± 0.04 ^a^	5.00 ± 0.04 ^a^	5.00 ± 0.04 ^a^	5.00 ± 0.04 ^a^
3	5.30 ± 0.12 ^a^	5.44 ± 0.01^c^	5.24 ± 0.12 ^a^	5.16 ± 0.05 ^a^	5.75 ± 0.04 ^b^	5.59 ± 0.05 ^b^
6	5.06 ± 0.07 ^a^	5.29 ± 0.03 ^b^	5.62 ± 0.10^c^	5.08 ± 0.03 ^a^	5.49 ± 0.03 ^b^	5.04 ± 0.03 ^a^
13	5.29 ± 0.08 ^a^	5.64 ± 0.04 ^c^	5.38 ± 0.08 ^b^	5.11 ± 0.04 ^a^	5.49 ± 0.06 ^b^	5.27 ± 0.04 ^a^
16	5.75 ± 0.03 ^b^	5.89 ± 0.05 ^d^	5.96 ± 0.10 ^d^	5.50 ± 0.10 ^b^	5.04 ± 0.05 ^a^	5.34 ± 0.06 ^a^
20	5.24 ± 0.04 ^a^	4.59 ± 0.03 ^a^	5.17 ± 0.01 ^a^	5.19 ± 0.03 ^a^	5.04 ± 0.05 ^a^	5.34 ± 0.05 ^a^
24	5.74 ± 0.03 ^b^	4.66 ± 0.05 ^a^	5.56 ± 0.03^c^	5.40 ± 0.03 ^b^	5.47 ± 0.04 ^b^	5.19 ± 0.02 ^a^
27	5.01 ± 0.05 ^a^	4.77 ± 0.10 ^a^	5.64 ± 0.04 ^b^	5.61 ± 0.04^c^	5.65 ± 0.03^c^	5.24 ± 0.06 ^a^
30	5.34 ± 0.04 ^a^	4.64 ± 0.11 ^a^	5.38 ± 0.04^c^	5.16 ± 0.03 ^a^	5.37 ± 0.03 ^b^	5.67 ± 0.06 ^b^

**Table 3:** *Penicillium* spp. during the storage of peanuts (Spanish type) at 4, 15, and 25 °C (log CFU/g), high inoculum conditions. The samples were inoculated with freshly produced spores (CS_0_) or with spores preliminarily stored at 4 °C for 30 days (SC_30_). For each column, the letters indicate significant differences among the sampling times; the letter a represents the lowest value, and the alphabetical order indicates the values’ increase (ANOVA and Tukey’s test, *p* < 0.05).

	25 °C		15 °C		4 °C	
Storage Time (Days)	CS_0_	CS_30_	CS_0_	CS_30_	CS_0_	CS_30_
0	5.00 ± 0.04 ^b^	5.00 ± 0.04 ^a^	5.00 ± 0.04 ^b^	5.00 ± 0.04 ^a^	5.00 ± 0.04 ^a^	5.00 ± 0.04 ^a^
3	5.20 ± 0.10 ^b^	5.34 ± 0.10 ^b^	5.42 ± 0.09 ^c^	5.06 ± 0.05 ^a^	5.97 ± 0.08 ^b^	5.50 ± 0.11 ^b^
6	5.19 ± 0.12 ^b^	5.05 ± 0.07 ^a^	4.88 ± 0.10 ^b^	5.08 ± 0.10 ^a^	5.39 ± 0.09 ^c^	5.40 ± 0.12 ^b^
13	5.84 ± 0.11 ^c^	4.99 ± 0.07 ^a^	4.82 ± 0.09 ^b^	5.20 ± 0.11 ^a^	5.52 ± 0.10 ^d^	5.46 ± 0.09 ^b^
16	5.19 ± 0.5 ^b^	4.90 ± 0.06 ^a^	4.65 ± 0.08 ^a^	5.40 ± 0.09 ^b^	5.52 ± 0.11 ^d^	5.35 ± 0.05 ^b^
20	5.93 ± 0.08 ^c^	5.78 ± 0.04 ^c^	5.29 ± 0.09 ^b^	5.10 ± 0.07 ^a^	5.52 ± 0.06 ^d^	5.35 ± 0.05 ^b^
24	5.28 ± 0.10 ^a^	5.27 ± 0.03 ^a^	4.34 ± 0.17 ^a^	5.50 ± 0.08 ^b^	5.52 ± 0.10 ^d^	5.47 ± 0.04 ^b^
27	4.67 ± 0.12 ^a^	5.36 ± 0.03 ^b^	5.33 ± 0.06 ^b^	5.60 ± 0.10 ^c^	5.37 ± 0.07 ^c^	5.81 ± 0.05 ^c^
30	4.92 ± 0.13 ^b^	5.46 ± 0.07 ^b^	5.25 ± 0.05 ^b^	5.20 ± 0.08 ^a^	5.67 ± 0.10 ^d^	5.52 ± 0.06 ^b,d^

**Table 4:** *Penicillium* spp. during the storage of peanuts (Virginia type) at 4, 15, and 25 °C (log CFU/g), low inoculum conditions. The samples were inoculated with freshly produced spores (CS_0_) or with spores preliminarily stored at 4 °C for 30 days (SC_30_). *, below the LOD (limit of detection, 2 log CFU/g). For each column, the letters indicate significant differences among the sampling times; the letter a represents the lowest value, and the alphabetical order indicates the values’ increase (ANOVA and Tukey’s test, *p* < 0.05).

	25 °C		15 °C		4 °C	
Storage Time (Days)	CS_0_	CS_30_	CS_0_	CS_30_	CS_0_	CS_30_
0	2.00 ± 0.00 ^a^	2.00 ± 0.00 ^a^	2.00 ± 0.00 ^a^	2.00 ± 0.00 ^a^	2.00 ± 0.00 ^a^	2.00 ± 0.00 ^a^
3	4.98 ± 0.01 ^c^	5.21 ± 0.03 ^c^	-	2.49 ± 0.02 ^a^	2.61 ± 0.01 ^b^	2.32 ± 0.03 ^a^
6	4.56 ± 0.01 ^b^	3.27 ± 0.01 ^b^	2.06 ± 0.08 ^a^	-	-	2.04 ± 0.05 ^a^
13	5.05 ± 0.04 ^c^	2.32 ± 0.03 ^a^	2.32 ± 0.03 ^a^	2.32 ± 0.03 ^a^	-	-
16	4.88 ± 0.00 ^c^	3.03 ± 0.01 ^b^	-	2.85 ± 0.01 ^b^	-	2.78 ± 0.01 ^b^
20	- *	3.63 ± 0.01 ^b^	-	-	2.04 ± 0.09 ^a^	2.78 ± 0.03 ^b^
24	-	3.52 ± 0.02 ^b^	2.06 ± 0.08 ^a^	-	-	2.32 ± 0.03 ^a^
27	-	-	-	-	-	-

**Table 5:** *Penicillium* spp. during the storage of peanuts (Spanish type) at 4, 15, and 25 °C (log CFU/g), low inoculum conditions. The samples were inoculated with freshly produced spores (CS_0_) or with spores preliminarily stored at 4 °C for 30 days (SC_30_). *, below the LOD (limit of detection, 2 log CFU/g). For each column, the letters indicate significant differences among the sampling times; the letter a represents the lowest value, and the alphabetical order indicates the values’ increase (ANOVA and Tukey’s test, *p* < 0.05).

	25 °C		15 °C		4 °C	
Storage Time (Days)	CS_0_	CS_30_	CS_0_	CS_30_	CS_0_	CS_30_
0	2.00 ± 0.00 ^a^	2.00 ± 0.00 ^a^	2.00 ± 0.00 ^a^	2.00 ± 0.00 ^a^	2.00 ± 0.00 ^a^	2.00 ± 0.00 ^a^
3	4.03 ± 0.01 ^d^	3.11 ± 0.01 ^b^	4.41 ± 0.02 ^d^	2.06 ± 0.08 ^a^	2.62 ± 0.02 ^b^	2.75 ± 0.01 ^b^
6	3.34 ± 0.03 ^c^	3.21 ± 0.03 ^b^	2.04 ± 0.05 ^a^	3.55 ± 0.01 ^c^	-	2.62 ± 0.02 ^b^
13	2.62 ± 0.02 ^b^	3.90 ± 0.01 ^b^	2.72 ± 0.03 ^b^	2.32 ± 0.03 ^a^	-	-
16	3.04 ± 0.05 ^c^	2.91 ± 0.01 ^b^	-	2.61 ± 0.01 ^b^	2.71 ± 0.02 ^b^	-
20	2.32 ± 0.03 ^a^	- *	-	-	2.70 ± 0.00 ^b^	-
24	3.05 ± 0.04 ^c^	3.15 ± 0.03 ^b^	3.13 ± 0.02 ^c^	3.37 ± 0.01 ^c^	2.49 ± 0.02 ^b^	3.39 ± 0.01 ^c^
27	-	-	-	-	-	-

**Table 6:** Model parameters for linear regression of time/temperature vs. spore count of *Penicillium* spp. after drying process.

	Coefficient	Standard Error	*t*-Test	*p*-Value	R^2^
Constant	13.994	1.983	7.056	0.001	
Temperature	−0.242	0.044	−5.533	0.003	0.876
Time	0.010	0.026	0.392	0.711	0.144

## Data Availability

Data will be made available upon request.
